# *Weizmannia coagulans* BC99 Enhances Intestinal Barrier Function by Modulating Butyrate Formation to Alleviate Acute Alcohol Intoxication in Rats

**DOI:** 10.3390/nu16234142

**Published:** 2024-11-29

**Authors:** Cheng Li, Shirui Zhai, Mengyao Duan, Li Cao, Jie Zhang, Yao Wang, Ying Wu, Shaobin Gu

**Affiliations:** 1 College of Food and Bioengineering, Henan University of Science and Technology, Luoyang 471023, China; lc15515735845@163.com (C.L.);; 2 National Demonstration Center for Experimental Food Processing and Safety Education, Luoyang 471023, China; 3 Henan Engineering Research Center of Food Microbiology, Luoyang 471023, China

**Keywords:** *Weizmannia coagulans*, acute alcohol intoxication, microbiota, butyrate, intestinal barrier

## Abstract

**Background/Objectives:** Probiotics have great potential in improving acute alcohol intoxication. The aim of this study was to investigate the mitigating effect and mechanism of action of *Weizmannia coagulans* BC99 on acute alcohol intoxication (AAI) in SD rats. **Methods:** BC99 was divided into different doses administered by gavage to rats, and a rat model of acute alcohol intoxication was established by multiple gavages of excess alcohol. **Results:** Our study demonstrated that *W. coagulans* BC99 intervention significantly prolonged the latency period of intoxication; significantly attenuated alcohol-induced lipid elevation, liver injury, hepatic inflammation, and intestinal barrier damage; and lowered plasma endotoxin (LPS) levels in rats. In addition, *W. coagulans* BC99 could effectively restore the balance of intestinal flora, increase the abundance of *Lachnospiraceae*_*NK4A136*, *Prevotellaceae*_*NK3B31*, *Parabacteroides*, and *Ralstonia*, and thus increase the content of intestinal short-chain fatty acids (SCFAs), especially butyric acid. Moreover, we demonstrated through sodium butyrate validation experiments that butyrate could attenuate intestinal barrier damage and reduce the diffusion of LPS, thereby reducing liver inflammation. **Conclusions:** In conclusion, *W. coagulans* BC99 ameliorates acute alcohol intoxication in rats by increasing the abundance of butyrate-producing genera and thereby increasing butyrate abundance to alleviate intestinal barrier injury.

## 1. Introduction

Acute alcohol intoxication (AAI) is an adverse phenomenon in which a single large dose of alcohol exceeds the body’s metabolic rate, resulting in high ethanol levels in the blood, leading to functional and metabolic disorders in multiple organs [1]. Most patients with acute alcohol intoxication show only transient behavioral and consciousness abnormalities, and the prognosis is good, but some of the patients may have neurological, circulatory, digestive, and other organ function damage or even respiratory and circulatory failure, which may be life-threatening. However, some patients may suffer from neurological, circulatory, gastrointestinal, and other organ function damage, or even respiratory and circulatory failure, which are life-threatening. According to epidemiologic statistics, about 2.5 million people die from alcoholism every year worldwide [2]. According to the Global Burden of Disease Study 2019, alcoholism is the ninth leading risk factor for disability-adjusted life years [3]. Current clinical medications (naloxone, metadoxine) are temporarily effective in patients with acute alcohol intoxication, but they have significant adverse effects and do not influence the pathologic progression of acute alcohol intoxication [4].

The pathogenesis of AAI involves various aspects of oxidative stress, lipid metabolism, and intestinal flora [5]. Among them, intestinal flora dysbiosis is one of the important factors contributing to AAI [6]. Research has indicated that excessive alcohol consumption changes the composition and quantity of gut microbiota, disturbs the balance of gut flora, raises the levels of Gram-negative bacteria, and generates high levels of endotoxin [7,8]. Under typical circumstances, the intestinal barrier serves to block endotoxin and other compounds from entering the bloodstream. However, ethanol consumption disrupts this barrier, causing an increase in intestinal permeability. This allows for a significant quantity of endotoxin to enter the liver via the portal vein, resulting in the activation of hepatic stellate cells and the occurrence of hepatic inflammation [9,10]. Therefore, controlling the equilibrium of gut microbiota and repairing the intestinal barrier could emerge as a promising treatment approach for AAI.

Probiotics are live microorganisms that, when given in sufficient quantities, will provide health benefits to the host [11]. Previous studies have shown that probiotics can inhibit hepatic inflammation, balance and restore the gut microbiota, and maintain intestinal barrier integrity [12,13], showing promising results in ameliorating alcoholic liver injury. In addition, probiotics such as *Lactobacillus plantarum* ZS62 and *Lactobacillus rhamnosus* GG have been shown to have significant ameliorative effects on alcoholic liver injury [14,15]. *W. coagulans* is a Gram-positive bacterium with rod-shaped cell morphology and the ability to form endospores; *W. coagulans* has considerable resistance to extreme temperatures, pH values, and choline compared to other probiotics [16]. *W. coagulans* can produce organic acids and bacteriocins, which can significantly improve gastrointestinal microecology and increase the number of beneficial intestinal microorganisms while antagonizing pathogenic microorganisms [17,18]. In addition, *W. coagulans* could inhibit the production of pro-inflammatory factor IL-8 and increase the production of the anti-inflammatory factor IL-10, with immunomodulatory effects [19]. The discovery of these probiotic properties suggested great potential in the treatment of AAI. Experimental studies have investigated the effect of probiotics in alleviating AAI, but the mechanism still needs to be studied in depth and systematically, and there has been no study related to the alleviation of AAI by *W. coagulans*. These findings indicate that *W. coagulans* has great potential in the treatment of AAI. Against this background, this study established a rat model of acute alcohol intoxication, and studied the resistance of *W. coagulans* BC99 to alcohol injury and its possible mechanism of combining with intestinal flora.

## 2. Materials and Methods

### 2.1. Strains and Preparation of Bacterial Suspensions

The *W. coagulans* 99 (hereinafter referred to as BC99) strain was obtained from Wecare Probiotics Co., Ltd. (Suzhou, China). Bacterial powder of BC99 contained 1 × 10^11^ CFU/g. A corresponding dose of bacterial suspension was obtained by dilution with sterile saline before gavage. After preparation, it was refrigerated at 4 °C as a spare.

### 2.2. Animal and Experimental Design

A total of 50 SPF-grade male SD rats, 4~6 weeks old, were acquired from SPF Biotechnology Co., Ltd. (Beijing, China). The animals were kept at room temperature (21–25 °C), a humidity of 48–55%, and a 12/12 h light/dark cycle. This study provided standardized feed and water, as well as a comfortable breeding environment, and regularly monitored the health status of rats to enhance their comfort. We referred to the Taheri [20] method and made improvements by using a randomized block design grouping method. Moreover, we determined a sample size of 10 animals per group based on the 3R principle for experimental animals and the Power Analysis Method [21]. A total of 50 male SD rats were randomly allocated into the negative control group (NC), model group (AD), low-dose BC99 group (AL), medium-dose group (AM), and high-dose group (AH), with 10 rats in each group. This study used Red star Erguotou with an alcohol content of 53%, purchased from Beijing Red Star Co., Ltd. (Beijing, China). Rat maintenance feed was purchased from SPF Biotechnology Co., Ltd.

**Figure 1:**
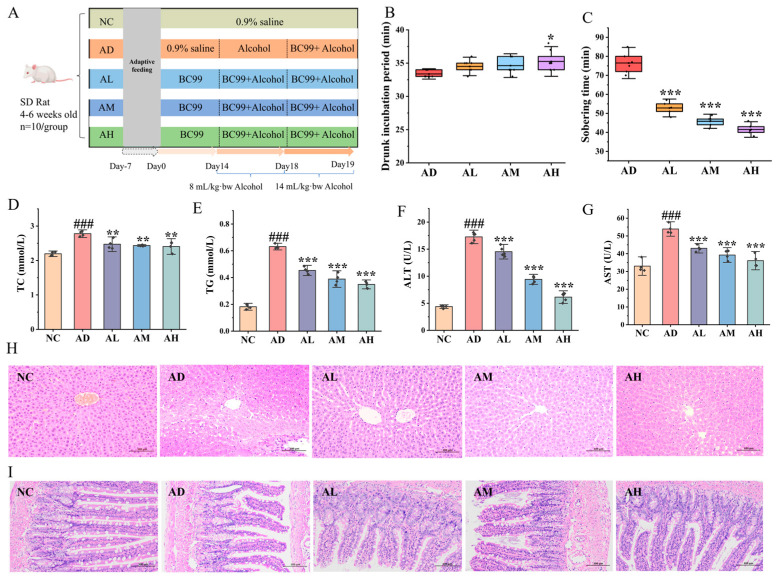
BC99 ameliorated hyperlipidemia and ileum and liver damage in AAI rats. (**A**) Experiment design, (**B**) drunk incubation period, (**C**) sobering time, (**D**) plasma TC, (**E**) plasma TG, (**F**) plasma ALT, (**G**) plasma AST, (**H**) liver H&E staining, magnification × 200, (**I**) ileum H&E staining, magnification × 200. ^###^ *p* < 0.001 vs. the NC group; * *p* < 0.05, ** *p* < 0.01, *** *p* < 0.001 vs. the AD group.

After a one-week acclimatization period, rats in the model and control groups were gavaged with saline at 9 a.m. every day, and the AL, AM and AH groups were gavaged with 10^6^, 10^7^, and 10^8^ CFU/g bw of BC99 bacterial solution according to the body weights of the rats every day for 19 days. Starting from the 15th day, the control group was gavaged with saline, and the model and BC99 groups were gavaged with white wine at a dose of 53% (*v*/*v*) 3.34 g/kg bw, and the dose of white wine was adjusted to 53% (*v*/*v*) 5.87 g/kg bw at the end of the last day of the experiment in order to cause an acute alcohol intoxication model. After fasting for 14 h, the rats were euthanized by injection of anesthetic and then executed, and blood, liver, ileum and cecum contents were collected for subsequent determination and analysis of relevant indexes. The experimental design is shown in Figure 1A. This experiment was accomplished under the approval of the Ethics Committee of Henan University of Science and Technology, China, animal license number [SYXK(Yu)2021-0011].

### 2.3. Overturned Reflex Test

After the last gavage, 6 rats from each group except the control group were tested. The rats were placed in a cage with their backs facing downward, and their postures were corrected to face downward every 5 s until the rats could not correct themselves immediately. When the rats could not turn over on their own for 30 s, the reflex was considered to have disappeared (intoxication); when the rats could turn over on their own again, the reflex was considered to have recovered (sobriety). The latency of intoxication (the time from the time of intoxication to the disappearance of the reflex) and the time of sleep (the time from the disappearance of the reflex to its recovery) were counted one by one after the administration of alcohol to the rats.

### 2.4. Measurement of Serological Indicators

Blood samples were centrifuged at 4 °C and 4000 r/min for 15 min to collect serum, and the levels of total cholesterol (TC), triglyceride (TG), alanine aminotransferase (ALT), and aspartate aminotransferase (AST) in the serum samples were measured. Kits to measure these were acquired from Nanjing Jiancheng Biotechnology Research Institute Co., Ltd. Lipopolysaccharide (LPS) was acquired from BIOISCO (Lianyungang, China) Biotechnology Co., Ltd.

### 2.5. Histopathological Analysis

Hematoxylin–eosin (HE) staining: The liver and ileum of rats were taken, immersed in 4% paraformaldehyde solution, and immobilized for 48 h. After the tissues were dehydrated and encapsulated in wax sections, they were stained with HE staining, and the histopathological changes were observed under a microscope.

### 2.6. Measurement of Liver Inflammatory Factors

Homogenates were prepared by adding an appropriate amount of liver tissue into a 9 times higher volume of normal saline in an ice water bath (4 °C, 12,000× *g*, 15 min). The levels of tumor necrosis factor-α (TNF-α), interferon-γ (IFN-γ), and interleukin-10 (IL-10) in the liver were detected with the supernatant solution. The kits to measure these were acquired from BYabscience, Nanjing, China.

### 2.7. Intestinal Barrier Function Assessment

The ileal tissue was excised and fixed in 4% paraformaldehyde solution. The samples were embedded in paraffin, sectioned, dewaxed, and hydrated. Following antigen retrieval, the endogenous peroxidase was blocked with 3% hydrogen peroxide, and non-specific antigens were blocked with serum at 37 °C for 30 min. After they were washed with PBS, the sections were incubated with anti-Occludin, anti-Claudin-1, and anti-ZO-1 antibodies overnight at 4 °C. Secondary antibodies were added after three rinses with PBS and incubated for 50 min at room temperature. A DAB assay kit was used for color development, and brown-yellow cells were positive. All slices were counterstained with hematoxylin. Three fields of view from each slice were randomly read and the average optical density was calculated using Image J v1.51w software.

### 2.8. Short-Chain Fatty Acid (SCFA) Determination

We took 0.20 g of fresh feces, added 1.60 mL of sterilized deionized water, mixed them well by shaking, let it stand at room temperature for 20 min, centrifuged the mixture at 4 °C 15,000 rpm for 15 min, transferred the supernatant to a new EP tube, added 1.6 mL of sterilized deionized water into the fecal precipitate again, repeated the above operation, mixed the supernatant with the combined supernatant, and then filtered it through a 0.22 μm filter and took 0.20 mL of supernatant for GC tests. After filtering through a 0.22 m filter, 0.20 mL of the supernatant, 0.70 mL of sterile water, and 0.10 mL of 100 μg/m *n*-butanol were used to prepare the sample for GC detection. The standard solution was formulated by incorporating six SCFA standards: acetic acid, propionic acid, isobutyric acid, butyric acid, isovaleric acid, and valeric acid. Then, 0.20 mL of mixed standard solution, 0.70 mL of sterile water, and 0.10 mL of *n*-butanol were added to the sample, so that the *n*-butanol sampling concentration was 100 μg/mL, using *n*-butanol as the reference, and the average of the peak area of *n*-butanol from several readings was taken to correct for the batch-to-batch error. Subsequently, the standard curve could be generated by testing it with the machine, using the concentration of the standard acid as the *x*-axis and the peak area as the *y*-axis.

The analytical conditions for gas chromatography were as follows: the separation column was JN-5MS (30 m× 0.25 mm, 0.25 μm), the inlet temperature was 250 °C, and the injection volume was 1 μL. The procedure of the heating program was as follows: starting temperature: 40 °C, held for 1 min; 8 °C/min to 60 °C, held for 1 min; 10 °C/min to 70 °C, held for 1 min; 20 °C/min to 220 °C, held for 10 min; constant flow 1.5 mL/min, no shunt.

### 2.9. 16S rRNA High-Throughput Sequencing

The DNA extraction kit was utilized to isolate the DNA from the intestinal microorganisms found in the cecum contents of SD rats, and the obtained DNA samples were quantitatively analyzed by a nucleic acid analyzer and detected by 10 g/L agarose gel electrophoresis. The samples with a DNA concentration greater than 50 mg/L and clear bands met the sequencing requirements. The samples were sent to BIOTREE biomedical technology Co., Ltd. (Shanghai, China), for V3–V4 variable region analysis of the bacterial 16SrDNA. The main experimental process is as follows: TransStartFastpfuDNA Polymerase (TransGen Biotech Co., Ltd., AP221-02, Beijing, China) was used for PCR, with 3 replicates for each sample. PCR products from the identical sample were combined and analyzed using 20 g/L agarose gel electrophoresis, followed by retrieval of the PCR products through gel cutting with the AxyPrepDNA gel recovery kit (Axygen, Union City, CA, USA). Subsequently, the samples were sequenced on an Illumina NovaSeq platform (Illumina, San Diego, CA, USA).

### 2.10. Sodium Butyrate Verification Experiment

Thirty rats were randomly allocated into control, model, and NaB groups, each comprising ten rats. The intervention method was the same as above. The dose for the sodium butyrate validation group was 0.3 mg/g bw. At the end of the experiment, ileal HE sections were observed, and tight junction proteins (ZO-1, Occludin, Claudin-10), LPS, hepatic inflammatory factors (TNF-α, IL-10, INF-γ), and serological indices (lactate dehydrogenase (LDH), total bile acid (TBA), Nanjing Jiancheng Biotechnology Research Institute Co., Ltd. (Nanjing, China) were determined as described above.

### 2.11. Data Analysis

Graphs were created using Origin 2021 software. Statistical analysis was performed using SPSS 25.0 software. Experimental data are presented as mean ± standard deviation (n = 6) with a 95% confidence interval. Between-group comparison: If the data meet the assumptions of normal distribution and homogeneity of variance, independent sample *t*-tests can be used. If these assumptions are not met, the Mann–Whitney U test can be used. Multiple-group comparison: If the data meet the assumptions of normal distribution and homogeneity of variance, one-way ANOVA analysis can be used. If the data do not meet the assumptions of ANOVA, the Kruskal–Wallis test can be used, with *p* < 0.05 indicating a difference, *p* < 0.01 indicating a significant difference, and *p* < 0.001 indicating a highly significant difference.

## 3. Result

### 3.1. BC99 Prolonged Intoxication Latency in AAI Rats

As shown in Figure 1B,C, the rats in the AD group had poor balance after drinking, the hind limbs were weak when crawling, they gradually entered into the intoxicated state, and the turn-right reflex disappeared. The rats in the AH group exhibited a 5.9% increase in the intoxication latency compared to the AD group (*p* < 0.05), with no significant difference observed in the other groups. However, the duration of sobriety in the low-, medium-, and high-BC99 groups was reduced by 30.9%, 40.1%, and 45.6%, respectively (*p* < 0.001), and it was positively correlated with the dose. In conclusion, BC99 could reduce the intoxication state and shorten the sobriety time of rats, but it was related to the intervention dose.

### 3.2. BC99 Improved Physiological Indexes in AAI Rats

The intake of large amounts of alcohol caused damage to the liver function of rats, as evidenced by elevated lipids and elevated serum aminotransferases [22]. As shown in Figure 1D–G, the levels of TC, TG, ALT, and AST in the serum were significantly elevated in rats in the AD group compared to those in the NC group (*p* < 0.001). In contrast, the serum levels of TC, TG, ALT, and AST were markedly reduced in rats in the BC99 group compared to the AD group (*p* < 0.05). In the AH group, the serum levels of TC were reduced by 13.4% (*p* < 0.01), TG by 44.7% (*p* < 0.001), ALT by 64.5% (*p* < 0.001), and AST by 33.1% (*p* < 0.001), as compared with the AD group.

### 3.3. BC99 Attenuated Histologic Damage in AAI Rats

To further investigate the function of BC99 in alleviating AAI rats, HE staining of the liver and ileum was performed. HE staining of the liver is shown in Figure 1H. The liver cells of rats in the NC group were neatly arranged, with clear contours, no degeneration or necrosis, and the nucleus was large and round. The dry shrinkage of the AD group was disorganized, with turbid and swollen stem cells, a large number of necrotic cells, and a sparse cytoplasm, which proved the presence of alcohol damage to the liver. The liver tissue morphology of rats supplemented with BC99, especially in the AH group, tended towards that of the NC group, with a reduced degree of cell necrosis, an orderly arrangement of hepatic cords, and liver cells radiating from the central vein to the periphery.

HE staining of the ileum is shown in Figure 1I. The ileum mucosal structure in the NC group was clear, the intestinal villi structure was normal, villous cells were arranged neatly and tightly, the mucosal epithelium was complete, and the structure of glandular cells in the lamina propria was clear; the intestinal villi of rats in the model group were obviously shortened and partially broken, the cells of the lamina propria were severely structurally disorganized with inflammatory cell infiltration, and the submucosal layer was congested and edematous, accompanied by inflammatory cell infiltration. The structure of rat ileum tissue in the BC99 group closely resembled that of the control group, with the intestinal villous epithelium remaining largely intact and a notable decrease in inflammatory cell infiltration compared to the model group.

### 3.4. BC99 Improved Inflammatory Response in AAI Rats

**Figure 2:**
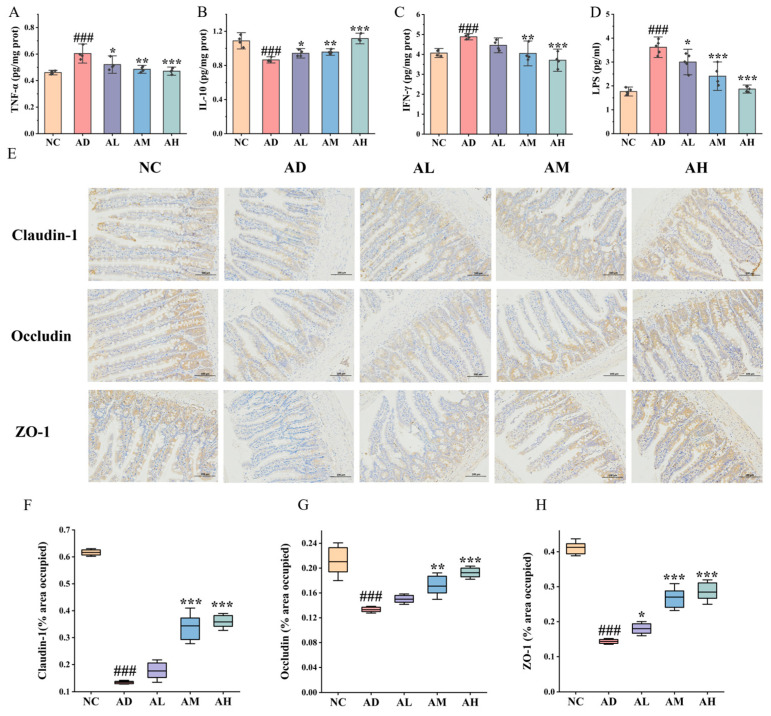
BC99 ameliorates the intestinal barrier and relieves liver inflammation in AAI rats. (**A**) Liver TNF-α, (**B**) liver IFN-γ, (**C**) liver TNF-α, (**D**) plasma LPS, (**E**) ileum IHC staining, magnification × 200, (**F**) ileum Claudin-1, (**G**) ileum Occludin, (**H**) ileum ZO-1. ^###^ *p* < 0.001 vs. the NC group. * *p* < 0.05, ** *p* < 0.01, *** *p* < 0.001 vs. the AD group.

Research found that inflammation is an important cause of acute alcohol intoxication injury. We further investigated the effects of BC99 supplementation on hepatic inflammatory factors in AAI rats. Overall, the expression of the inflammatory cytokines TNF-α and IFN-γ in the AD group showed a 30.4% and 20.2% increase, respectively, compared to that in the NC group (*p* < 0.001). The levels of the inflammatory cytokines TNF-α and IFN-γ in the BC99 group were markedly lower than those in the AD group, while the levels of inflammatory cytokines in the AH group were similar to those in the NC group. Furthermore, the level of the anti-inflammatory factor IL-10 in the liver of rats in the AD group decreased by 20.2% compared to the NC group (*p* < 0.001). The administration of BC99 treatment led to a significant elevation in the IL-10 level compared to the AD group, and the degree of increase was positively associated with the dosage (Figure 2A–C).

### 3.5. BC99 Improved Intestinal Barrier Function in AAI Rats

The intestinal barrier is the first line of defense for the body to prevent pathogenic microorganisms and their products from entering the systemic circulation, while alcohol abuse can compromise the integrity of the intestinal barrier. Therefore, in order to study the protective effect of *W. coagulans* on the intestinal barrier, serum LPS levels were measured. The AD group showed a 105% increase in serum levels of LPS compared to the NC group (*p* < 0.001), indicating that alcohol disrupted the intestinal barrier in rats, resulting in the leakage of LPS. Notably, LPS levels were significantly reduced by 48.4% (*p* < 0.001) in rats in the AH group compared to the AD group. This suggested that supplementation with BC99 enhances the intestinal barrier in the ethanol-induced AAI model (Figure 2D).

To further explore the role of BC99 in maintaining ileal intestinal barrier integrity, we examined ileal tight junction (TJ) proteins. The expression levels of the TJ proteins Occludin, Claudin-1, and ZO-1 were downregulated by 78.1%, 36.7%, and 65.1%, respectively, in AD rats compared with the NC group (*p* < 0.001). Occludin, Claudin-1, and ZO-1 expression were upregulated in the AH group compared to the AD group by 165.2%, 45.1%, and 97.9%, respectively (*p* < 0.001) (Figure 2E–H). In summary, BC99 decreased serum endotoxin levels, increased TJ protein expression, and enhanced intestinal barrier function in AAI rats.

### 3.6. BC99 Adjusted the Structure of Intestinal Flora in AAI Rats

**Figure 3:**
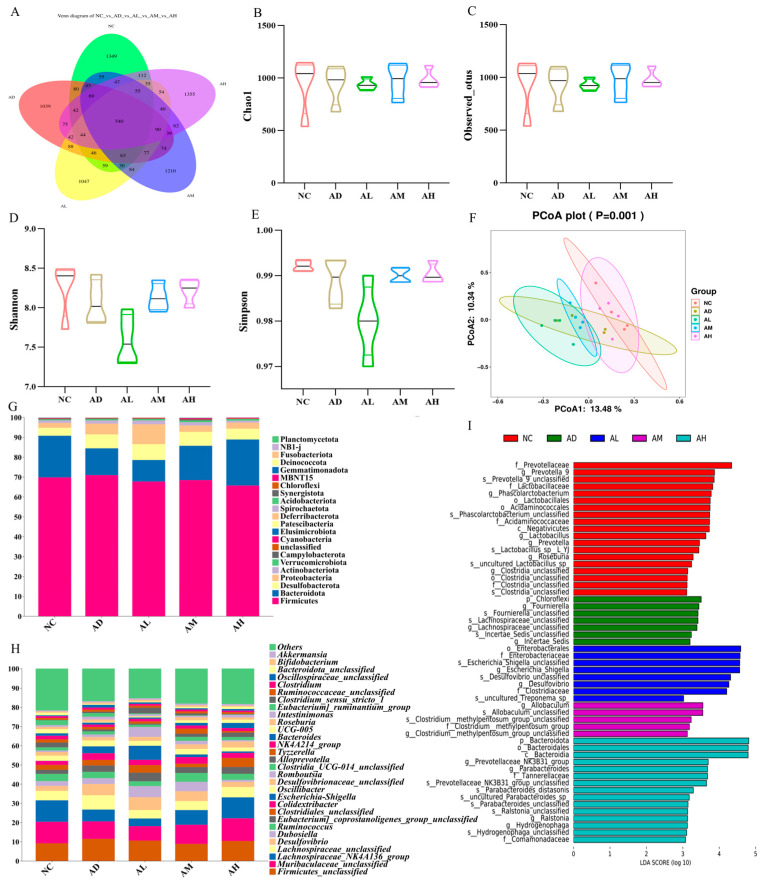
BC99 regulated the composition of gut microbiota. (**A**) Venn analysis, (**B**) Chao1 index, (**C**) observed index, (**D**) Shannon index, (**E**) Simpson index, (**F**) principal coordinate analysis of Bray–Curtis distance, (**G**) abundance of phylum-level flora, (**H**) abundance of genus-level flora, (**I**) analysis of differences in the microbial taxa by LEfSe.

We further investigated the effect of BC99 on the composition of the gut microbiota in alcohol-treated rats by using 16S rDNA gene sequencing of cecum content samples. Each de-emphasized sequence generated after noise reduction using DADA2 was called amplified sequence variants (ASVs), which was equivalent to clustering at 100% similarity, and a larger number of ASVs indicates greater diversity in the microbial community. The Venn diagram of ASVs was plotted, as shown in Figure 3A; the unique numbers of ASVs in NC, AD, AL, AM, and AH were 1349, 1039, 1047, 1210, and 1355, respectively, while the number of ASVs common to each group was 540. The results indicated that alcohol decreases the diversity of rat intestinal flora, and the difference between the number of ASVs in each group indicated that there was a difference in the similarity of rat intestinal flora in each group.

The Chao and Observed_otus indices reflected community richness and Shannon and Simpson indices reflected community diversity. In Figure 3B–E, it can be observed that the diversity index decreased in the AD group compared to the NC group, indicating that alcohol had an impact on the intestinal flora of rats. Different doses of BC99 had varying effects on community richness. The AM and AH groups showed an increase in the diversity of the intestinal flora compared to the AD group, but the difference was not statistically significant. Principal coordinate analysis (PCoA) based on Bray–Curtis distance revealed distinct differences in the intestinal microbiota composition between rats in the AD and NC groups. In addition, the composition of the gut microbiota in the AH group was markedly distinct from that in the AD group, indicating that the addition of BC99 had a significant impact on the gut microbiota of AAI rats. Moreover, the overall composition of the gut flora in the AH group was significantly more similar to that in the NC group compared to the AD group (Figure 3F).

The gut microbiota of all experimental groups showed a similar phylogenetic structure (Figure 3G). The intestinal microbiota was mainly composed of Firmicutes, Bacteroidetes, Desulfovibrio, Proteobacteria, and Actinomycetes. Compared with the NC group, the rats in the AD group showed a significant decrease in the level of Bacteroidetes and a significant increase in Desulfovibrio, Proteobacteria, and Actinomycetes, suggesting that the phylum level of the intestinal flora of the rats with acute alcohol intoxication was significantly altered. After being exposed to moderate and high levels of BC99, the level of Bacteroidetes significantly increased, while the Desulfovibrio, Proteobacteria, and Actinomycetes levels decreased, and the trend was positively correlated with the dose, with the composition of the phylum-level colonies in the AH and NC groups being the closest to each other.

At the genus level (Figure 3H), *Firmicutes*_*unclassified*, *Muribaculaceae*_*unclassified*, and *Lachnospiraceae*_*NK4A136* were found to comprise the largest percentage of intestinal biota in all groups. Compared to the NC group, the AD group showed a significant decrease in the abundance of *Lachnospiraceae*_ *NK4A136*, while the abundance of *Desulfovibrio*, *Lachnospiraceae*_*unclassified*, *Colidextribacter*, and *Escherichia*-*Shigella* significantly increased. Relative to the AD group, *Dubosiella*, *Escherichia*-*Shigella*, and *Romboutsia* became dominant genera in the AL group, and *Lachnospiraceae*_*NK4A136*, *Dubosiella* in the AM group. *Ruminococcus* increased in abundance, and *Desulfovibrio* and *Escherichia*-*Shigella* decreased in abundance. The abundance of *Lachnospiraceae*_*NK4A136* and *Alloprevotella* in the AH group was significantly increased, the abundance of *Desulfovibrio*, *Colidextribacter*, and *Escherichia*-*Shigella* was decreased, and *Lachnospiraceae*_*NK4A136* became the dominant group in the AH group. The results indicated that the composition of the microbiota gradually returned to that of the NC group after BC99 treatment.

Using LEfSe analysis, it was possible to identify microbial taxa that differed significantly in AAI rats after BC99 supplementation (Figure 3I). *Fournierella* was found to be enriched only in the AD group. The dominant group in the AL group was *Enterobacterales*, the dominant group in the AM group was *Allobaculum*, and the differential genera in the AH group were *Ralstoniaa Prevotellaceae*_*NK3B31* and *Parabacteroides*, while the control group exhibited the highest abundance of _*Prevotella*_*9*, *Phascolarctobacterium*, and *Lactobacillaceae*.

**Figure 4:**
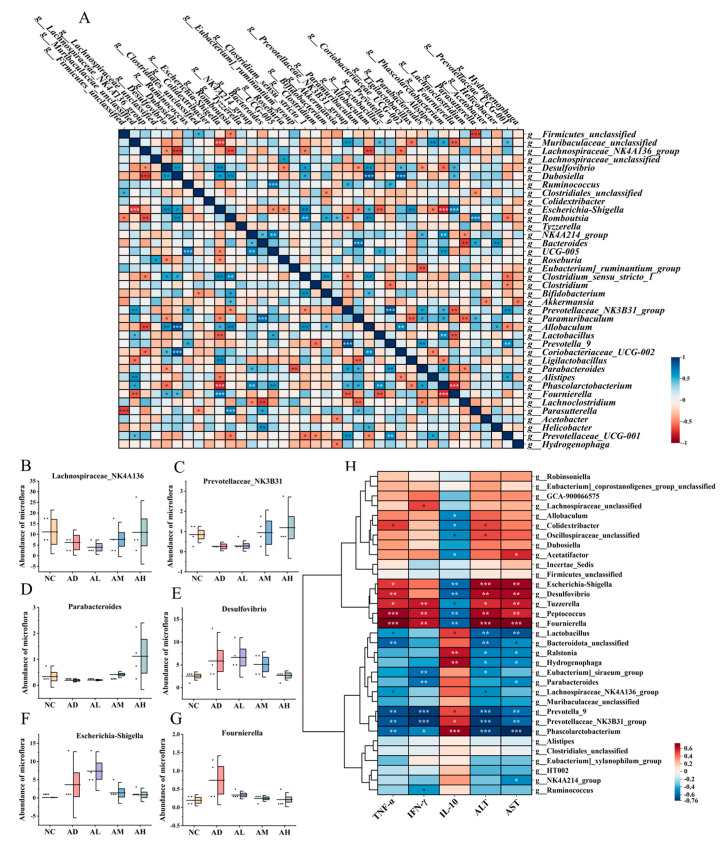
BC99 elevated the levels of beneficial bacteria while reducing the levels of pathogenic bacteria. (**A**) Analysis of flora interaction, (**B**) *Lachnospiraceae_NK4A136* abundance, (**C**) *Prevotellaceae*_*NK3B31* abundance, (**D**) *Parabacteroides* abundance, (**E**) *Desulfovibrio* abundance, (**F**) *Escherichia*-*Shigella* abundance, (**G**) *Fournierella* abundance, (**H**) correlation analysis of intestinal flora with inflammation and physiological indexes. In (**A**,**H**), “*, **, ***” represented significance, * *p* < 0.05, ** *p* < 0.01, *** *p* < 0.001. In (**B**–**G**), “*” represented the distribution of sample points.

In order to study the relationship between the flora, flora interaction analysis was conducted in this study (Figure 4A). The results showed that *Desulfovibrio* was significantly positively correlated with *Escherichia*-*Shigella* and *Fournierella*, and significantly negatively correlated with *Lachnospiraceae*_*NK4A136* and *Parabacteroides*. In addition, *Romboutsia* was significantly negatively correlated with *Parabacteroides* and *Lachnospiraceae*_*NK4A136*. *Prevotellaceae*_*NK3B31* was significantly positively correlated with *Parabacteroides* and *Prevotella*_*9*. This proved that alcohol intake leads to reciprocal growth of pathogenic bacteria, whereas BC99 intervention led to a common increase in the abundance of beneficial intestinal bacteria and also inhibited the colonization of pathogenic bacteria, as evidenced by the changes in the abundance of the different flora in Figure 4B–G.

These results suggested that BC99 altered gut microbial composition, resulting in an enrichment of beneficial bacteria and a decrease in pathogenic bacteria, and that intervention with BC99 at high doses skewed the colony composition of the AH group to be more similar to the control group. However, 16S rRNA gene sequencing did not identify BC99 in the feces of the BC99-treated group, which may be due to the limited colonization capacity of the bacteria.

### 3.7. Correlation Analysis of Bacterial Flora with Inflammation and Liver Function Indicators

The key strains of BC99 for alleviating acute alcohol intoxication in rats were explored by correlating inflammatory factors and liver function indices with gut microorganisms at the genus level (Figure 4H). In this study, *Desulfovibrio*, *Escherichia*-*Shigella*, *Fournierella*, and *Tyzzerella* were found to be significantly positively correlated with pro-inflammatory factors (TNF-α, INF-γ), ALT, and AST, and significantly negatively correlated with the anti-inflammatory factor IL-10. This indicated that they were strongly correlated with the occurrence of the inflammatory response and liver injury in AAI rats. In addition, *Lachnospiraceae*_*NK4A136*, *Prevotellaceae*_*NK3B31*, *Parabacteroides*, *Ralstoniaa*, and *Hydrogenophaga* in the BC99 group showed a significant positive correlation with anti-inflammatory factors and a significant negative correlation with TNF-α, INF-γ, ALT, and AST, suggesting that they may be the key strains of BC99 in alleviating acute alcohol intoxication.

### 3.8. BC99 Improves SCFA Production in AAI Rats

**Figure 5:**
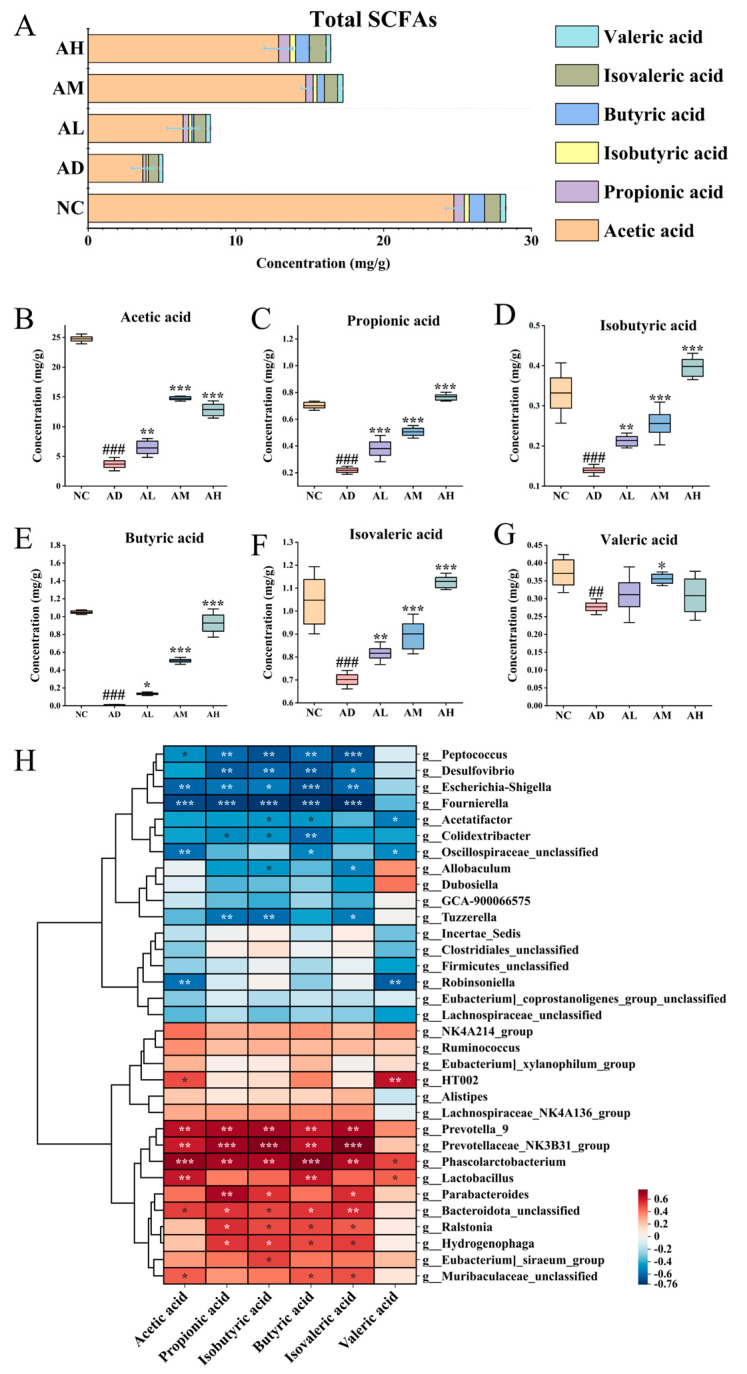
BC99 regulated the SCFA concentration. (**A**) Total SCFAs, (**B**) concentration of acetate acid, (**C**) concentration of propionic acid, (**D**) concentration of isobutyric acid, (**E**) concentration of butyric acid, (**F**) concentration of isovaleric acid, (**G**) concentration of valeric acid, (**H**) correlation analysis of intestinal flora with SCFAs. ^##^ *p* < 0.01, ^###^ *p* < 0.001 vs. the NC group. * *p* < 0.05, ** *p* < 0.01, *** *p* < 0.001 vs. the AD group.

In order to study the effect of BC99 supplementation on SCFA production in more detail, we measured the concentrations of acetate, propionate, isobutyrate, butyrate, isovalerate, and valerate in feces (Figure 5A–G). Alcohol significantly decreased the content of the above six SCFAs (*p* < 0.001). SCFAs were significantly restored after BC99 intervention, and the concentrations of the SCFAs were positively correlated with the dose of BC99, except for acetate and valerate. The results indicated that BC99 could significantly restore the levels of SCFAs in the intestine compared with the AD group.

Correlation analysis was performed between intestinal flora and SCFAs at the genus level to explore the key strains of BC99 that mediate the restoration of SCFA contents in AAI rats (Figure 5H). *Desulfovibrio*, *Peptococcus*, *Fournierella*, and *Tyzzerella* were found to be significantly negatively correlated with SCFAs, which demonstrated that the reduction in SCFAs was inevitable when the abundance of these bacteria was elevated in the intestinal tract of AAI rats. In addition, *Prevotellaceae*_*NK3B31*, *Parabacteroides*, *Ralstoniaa*, and *Hydrogenophaga* showed a significant positive correlation with SCFAs. The results demonstrate that the elevated concentration of butyric acid caused by such bacteria may be critical in alleviating acute alcohol intoxication in rats.

### 3.9. Sodium Butyrate Enhances Intestinal Barrier Function

**Figure 6:**
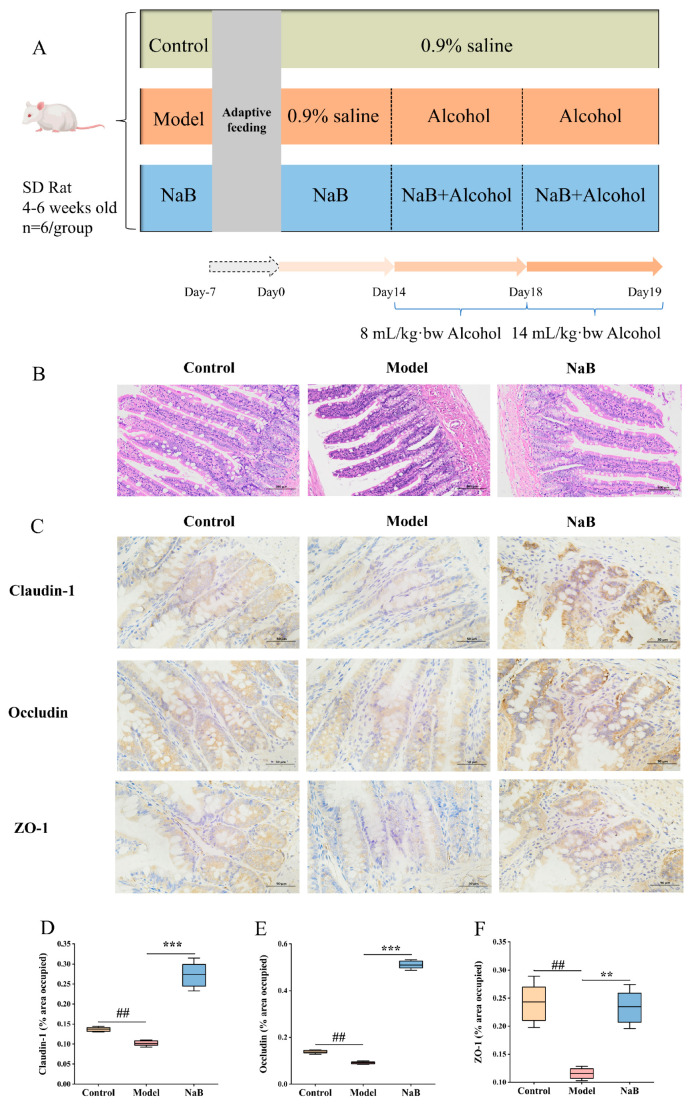
Sodium butyrate strengthened the intestinal barrier. (**A**) Experimental design, (**B**) ileum H&E staining, magnification × 200, (**C**) ileum IHC staining, magnification × 400, (**D**) ileum Claudin-1, (**E**) ileum Occludin, (**F**) ileum ZO-1. ^##^ *p* < 0.01 vs. the NC group. ** *p* < 0.01, *** *p* < 0.001 vs. the AD group.

To investigate whether the improvement of acute alcohol intoxication by BC99 is associated with an increase in butyrate-producing beneficial bacteria, a validation experiment using sodium butyrate was conducted in this study (Figure 6A). HE staining showed that NaB could restore the obvious disorder of villi structure in the ileum caused by alcohol and reduce the infiltration of inflammatory factors in the lamina propria (Figure 6B). Immunohistochemistry was employed to assess the levels of Occludin, Claudin-1, and ZO-1 TJ proteins in the intestinal tissues (Figure 6C–F). The findings indicated a decrease in the expression of Occludin, Claudin-1, and ZO-1 proteins in the ileum tissues of rats in the experimental group (*p* < 0.01). The NaB intervention group showed increased expression levels of the TJ proteins Occludin, Claudin-1, and ZO-1 compared to the control group (*p* < 0.001). These findings indicated that NaB enhanced the maintenance of the intestinal barrier in AAI rats by upregulating the expression of TJ proteins in the intestines.

**Figure 7:**
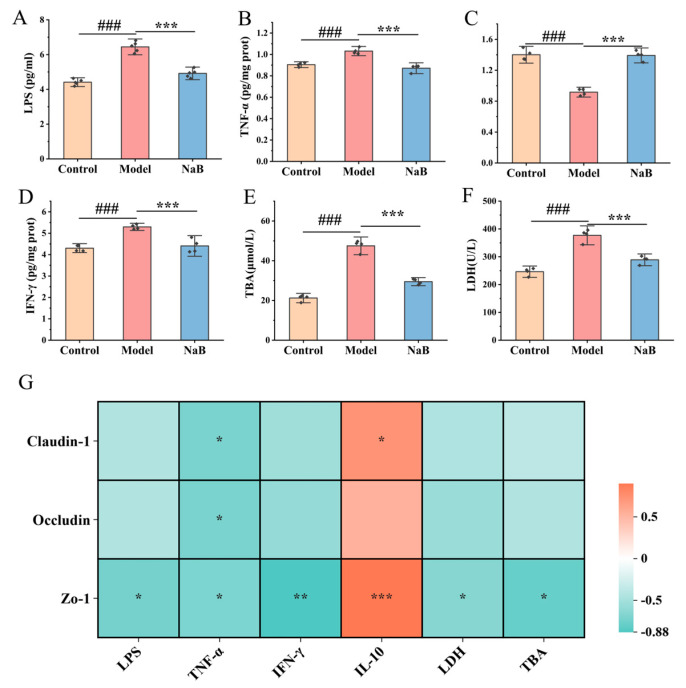
Sodium butyrate alleviated liver inflammation. (**A**) Plasma LPS, (**B**) liver TNF-α, (**C**) liver IFN-γ, (**D**) liver IL-10, (**E**) plasma TBA, (**F**) plasma LDH, (**G**) correlation analysis between inflammatory factors and intestinal TJ proteins. ^###^ *p* < 0.001 vs. the NC group. * *p* < 0.05, ** *p* < 0.01, *** *p* < 0.001 vs. the AD group.

Compared to the control group, the model group showed significantly elevated levels of plasma LPS, LDH, and TBA (*p* < 0.001). In contrast, the NaB intervention model group exhibited decreased levels of plasma LPS, LDH, and TBA compared to the model group (*p* < 0.001) (Figure 7A,E,F). Compared to the control group, levels of TNF-α and IFN-γ were found to be higher and IL-10 was found to be lower in the livers of rats in the model group (*p* < 0.001). In comparison, levels of TNF-α and IFN-γ were lower and IL-10 was higher in the livers of rats in the NaB intervention model group (*p* < 0.001) (Figure 7B–D).

Correlation analysis revealed a negative association between the levels of pro-inflammatory factors in the liver and the expression of the intestinal TJ proteins Occludin, Claudin-1, and ZO-1, and a significantly positive correlation with anti-inflammatory factors (*p* < 0.05). Studies have demonstrated that alcohol-induced elevation of intestinal permeability ultimately resulted in hepatic inflammation (Figure 7G). These results indicate that sodium butyrate significantly increases the expression of intestinal Occludin, Claudin-1, and ZO-1 proteins, improves intestinal permeability, and reduces serum LPS levels, thus reducing liver inflammation and improving alcohol-induced hepatic function impairment.

## 4. Discussion

Acute alcohol intoxication typically refers to a range of physical harm resulting from one-time heavy alcohol consumption, predominantly resulting in symptoms such as lack of coordination, decreased breathing, and unconsciousness [23]. Currently, medications for acute alcohol intoxication, like naloxone and metadoxine, can accelerate the metabolism of alcohol in the body to facilitate alcohol detoxification, but the majority of synthetic drugs have harmful side effects. Intestinal microecological dysregulation and impaired intestinal barrier integrity are important factors in the pathogenesis of acute alcohol intoxication. The intestine is connected to the liver through the bile ducts and portal vein, and LPS can enter the liver directly through the intestine–liver axis and activate the pro-inflammatory signaling pathway TLR4, leading to the release of pro-inflammatory cytokines, thus amplifying the inflammatory response of AAI [24,25]. In previous studies, in an alcohol-induced AAI model in rats, the intoxication latency was shortened, the sobriety time was prolonged, the levels of ALT, AST, TC, and TG were increased, the hepatic levels of inflammatory factors were elevated, and the levels of anti-inflammatory factors were decreased, suggesting that alcohol leads to the injury of hepatic tissues. The intervention of BC99 prolonged the intoxication latency and shortened the sobriety time of rats, and the levels of ALT, AST, TC, and TG were significantly reduced, hepatic pro-inflammatory factor levels were reduced, and anti-inflammatory factor levels were elevated, indicating that BC99 had a favorable ameliorative effect on AAI.

The intestinal barrier serves as the body’s primary defense mechanism against the invasion of pathogenic microorganisms and their products into the body’s circulation, and alcohol abuse can disrupt ileum intestinal barrier integrity [26]. Impairment of the epithelial barrier due to disruption of intestinal epithelial TJ proteins, which create intracellular spaces between neighboring epithelial cells and allow the passage of pathogens, is a prominent factor in the pathogenesis of AAI [27,28]. In this study, we analyzed the expression levels of TJ proteins in the ileum by immunohistochemistry. Compared with the NC group, the ileum TJ protein expression levels were significantly reduced in the AD group of rats, which indicated that alcohol disrupts intestinal barrier function, and BC99 restored the expression levels of the TJ proteins. We also examined the LPS content in the blood, which was in line with the suppression of the impaired intestinal barrier function of the AD group. The LPS content of the AD group was significantly elevated, and BC99 intervention reduced the LPS content. This outcome validated the ability of BC99 to modulate intestinal permeability and reinstate intestinal barrier function. Previous studies have shown that supplementation with *W. coagulans* can upregulate the expression of TJ proteins and mucins and restore the intestinal barrier function [29,30]. This is consistent with our research.

To understand the process by which *W. coagulans* mitigates the slowing of alcohol-induced intestinal and hepatic injury, we further analyzed the gut microbiota. Gut microecological imbalance can be characterized by quantitative and qualitative changes in the intestinal flora [31]. The prevalence of Bacteroidota was notably reduced in the gut of individuals with alcoholic fatty liver disease [32], while the prevalence of Proteobacteria was markedly elevated [33]. In contrast, in the present study, the abundance of Bacteroidota decreased, while the abundance of Desulfobacterota and Proteobacteria increased in the AD group compared to the NC group. BC99 intervention could reverse the changes in the bacterial flora, which was similar to the NC group. Aspergillus phylum is a conditionally pathogenic bacterium, and its increased abundance can be a sign of intestinal dysfunction and intestinal flora dysbiosis is often transferred from the intestinal tract to other tissues or organs of the organism, resulting in other diseases [34].

At the genus level, alcohol resulted in an increased abundance of *Desulfovibrio* and *Escherichia*-*Shigella*. *Desulfovibrio* belongs to the phylum Ascomycota, a class of sulfate-reducing H_2_S-producing anaerobes, and endogenous H_2_S activates a variety of channels (such as Ga^2+^ channels and TRP channels), damaging intestinal epithelial cells [35]. This demonstrated that damage to the intestinal epithelial barrier was associated with the upregulation of *Desulfovibrio* abundance. *Escherichia*-*Shigella* triggers inflammation in the intestines by infiltrating epithelial cells, inducing macrophage apoptosis, and releasing IL-1β, which is positively correlated with the level of intestinal inflammatory factors [36]. BC99 intervention restored the abundance of *Lachnospiraceae*_*NK4A136*, elevated the abundance of *Bacteroides*, and decreased the abundance of *Desulfovibrio* and *Escherichia*-*Shigella. Lachnospiraceae*_*NK4A136* is generally considered to be associated with improved intestinal function [37], produces a variety of SCFAs, especially butyric acid, to uphold and regulate the equilibrium of the host’s gut microbiota [38], and has been negatively correlated with a wide range of metabolic diseases and chronic inflammation [39]. In the flora interaction analysis, beneficial and pathogenic bacteria were shown to be antagonistic. BC99 increased the presence of beneficial bacteria while reducing the presence of pathogenic bacteria. In addition, in the Lefse analysis, the BC99 group was enriched with several other SCFA-producing bacteria such as *Prevotellaceae_NK3B31*, *Parabacteroides*, *Ralstonia*, etc. *Prevotellaceae_NK3B31* increased the levels of beneficial and pathogenic bacteria, played a role in protecting the intestinal mucosal barrier, improved intestinal function, and alleviated the inflammatory response [40]. It has also been demonstrated that Parabacteroides has a prominent role in the repair of intestinal mucosa [41]. *Ralstoniaa* species are butyric acid-producing bacteria, which play a prominent role in regulating intestinal permeability and preventing intestinal microecological imbalance [42,43].

In order to understand the role played by SCFAs in the alleviation of acute alcohol intoxication by BC99, the present study measured SCFAs in the feces of rats and made a correlation analysis between SCFAs and intestinal flora. The results showed that alcohol significantly decreased the content of six SCFAs in rats, and BC99 significantly restored the content of six SCFAs. There was a significant positive correlation between bacteria in the BC99 group and SCFAs. In fact, SCFAs are synthesized by the gut microbiota and play a critical role in preserving the integrity of the intestinal barrier; in particular, butyrate can enhance the intestinal barrier function [44]. Butyrate can be used as an energy donor for intestinal epithelial cells, which can phosphorylate the α-subunit of AMPK threonine 172 (Thr172) by altering the intracellular levels of AMP and ATP or activating CaMKKβ through regulating the intracellular concentration of Ca^2+^, which in turn activates AMPK [45,46], enhances the expression of TJ proteins in the intestinal epithelium, and ensures intestinal mechanical barrier integrity. Meanwhile, butyrate can act as an HDAC inhibitor [47], inhibit the activation of NLRP3 inflammasomes, reduce the level of cellular inflammatory cytokines, enhance the TJs between intestinal epithelial cells, and defend the integrity of the intestinal mechanical barrier [48].

To determine whether BC99 boosts the intestinal barrier through an increase in the prevalence of beneficial bacteria that produce butyric acid, thus alleviating acute alcohol intoxication in rats, we performed a sodium butyrate validation experiment. In this experiment, NaB was used to intervene in the AAI rat model. It was found that the structure of intestinal villi was significantly improved compared with the model group, and the expression of intestinal TJ proteins (Claudin-1, Occludin, ZO-1) was significantly elevated, which indicated that NaB has the ability to improve the function of the intestinal barrier, and that the intestinal-derived LPS translocation played a prominent role in ethanol-induced chronic inflammation of the liver [49]. In this study, LPS, liver injury indicators (LDH, TBA), and inflammatory factors (TNF-α, IFN-γ, IL-10) were detected in rats. The results showed that LPS, TNF-α, IFN-γ, LDH, and TBA were raised and IL-10 was decreased in rats of the model group. And LPS, TNF-α, IFN-γ, LDH, and TBA decreased and IL-10 increased in the NaB group. On this basis, we correlated TJ proteins with LPS, indicators of liver injury, and inflammatory factors. The results revealed that TJ proteins were negatively correlated with the expression of LPS, TNF-α, IFN-γ, LDH, and TBA, indicating that the increase in intestinal permeability caused harmful substances in the intestines to enter the liver through the intestinal–hepatic axis, which led to the aggravation of hepatic inflammatory reaction and damage to the liver. After the administration of NaB, the TJ proteins increased and the expression of inflammatory factors decreased in the model group. In conclusion, NaB inhibits the inflammatory response in AAI rats by upgrading the permeability of the intestinal barrier and reducing the entry of intestinal LPS into the liver via the gut–hepatic axis.

## 5. Conclusions

In this study, early intervention was performed with BC99, followed by multiple episodes of alcohol overdose-induced acute alcohol intoxication in rats. We found that supplementation with *W. coagulans* BC99 significantly altered the composition of the gut microbiota. In addition, *W. coagulans* BC99 particularly promoted elevated abundance of *Lachnospiraceae*_*NK4A136*, *Prevotellaceae_NK3B31*, *Parabacteroides*, and *Ralstonia*, which had been shown to be associated with the synthesis of SCFAs, especially butyric acid. The increase in butyrate could improve the expression of intestinal TJ proteins to influence intestinal permeability, thereby reducing the inflammatory response mediated by intestinal-derived LPS through the gut–hepatic axis into the liver, and ultimately alleviating acute alcohol intoxication in rats. The trial provided a theoretical basis for the use of probiotics as a pharmacological alternative for the treatment of acute alcohol intoxication.

We believe that this article still has some limitations. For example, although there is literature proving that propionate has a protective effect on the intestinal barrier, this article does not provide experimental evidence. Also, although BC99 has a relieving effect on acute alcohol poisoning in rats, the conclusions from animal experiments cannot be fully applied to clinical research, and further exploration is still needed.

## Data Availability

The original contributions presented in the study are included in the article; further inquiries can be directed to the corresponding author.
